# Design of the Cooperative Actuation in Hybrid Orthoses: A Theoretical Approach Based on Muscle Models

**DOI:** 10.3389/fnbot.2019.00058

**Published:** 2019-07-31

**Authors:** Francisco Romero-Sánchez, Javier Bermejo-García, Jorge Barrios-Muriel, Francisco J. Alonso

**Affiliations:** Department of Mechanical Engineering, Energy and Materials, University of Extremadura, Badajoz, Spain

**Keywords:** hybrid orthosis, functional electrical stimulation, inverse dynamics analysis, muscle model, biomechanics, rehabilitation, gait, fatigue

## Abstract

Hybrid orthoses or rehabilitation exoskeletons have proven to be a powerful tool for subjects with gait disabilities due to their combined use of electromechanical actuation to provide motion and support, and functional electrical stimulation (FES) to contract muscle tissue so as to improve the rehabilitation process. In these devices, each degree of freedom is governed by two actuators. The main issue arises in the design of the two actuation profiles for there to be natural or normative gait motion in which the two actuators are transparent to each other. Hybrid exoskeleton control solutions proposed in the literature have been based on tracking the desired kinematics and applying FES to maintain the desired motion rather than to attain the values expected for physiological movement. This work proposes a muscle-model approach involving inverse dynamics optimization for the design of combined actuation in hybrid orthoses. The FES profile calculated in this way has the neurophysiological meaningfulness for the device to be able to fulfill its rehabilitative purpose. A general scheme is proposed for a hybrid hip-knee-ankle-foot orthosis. The actuation profiles, when muscle tissue is fatigued due to FES actuation are analyzed, and an integrated approach is presented for estimating the actuation profiles so as to overcome muscle peak force reduction during stimulation. The objective is to provide a stimulation profile for each muscle individually that is compatible with the desired kinematics and actuation of the orthosis. The hope is that the results may contribute to the design of subject-specific rehabilitation routines with hybrid exoskeletons, improving the exoskeleton's actuation while maintaining its rehabilitative function.

## 1. Introduction

Spinal cord injury (SCI) and other neurological disorders impair the lower limbs' motor and sensory functions. The main treatment used to stop muscle atrophy is the use of robot assisted gait training devices. Currently there are many developments of active orthotics and exoskeletons under way, and some are already being commercialized. Several authors have reported on the state of the art of active orthoses and exoskeletons, see for example Aliman et al. (2017), on their control strategies (Jimenez-Fabian and Verlinden, 2012), or on the perspectives of their use (Herr, 2009; Young and Ferris, 2017). Assistance strategies for the most relevant active exoskeletons may be found in Yan et al. (2015).

Devices that combine functional electrical stimulation (FES) with active orthoses to assist gait, known as hybrid orthoses or FES-robot devices, have emerged as a promising technology in gait rehabilitation. The use of active orthoses in combination with FES is an effective strategy to optimize the outcomes of gait rehabilitation training. Using hybrid orthosis to stimulate the lower extremity muscles has proven to evoke muscle hypertrophy, increase strength, improve cardiopulmonary fitness, and reduce fatigue during gait, even in subjects with severe spasticity (Nightingale et al., 2007; Qiu and Taylor, 2016; Deley et al., 2017; Ekelem and Goldfarb, 2018; Lambach et al., 2018). Complete recent reviews of hybrid exoskeletons can be found in Stewart et al. (2017) for the upper limbs, and Anaya et al. (2018) for the lower limbs.

The correct use of FES in a hybrid orthosis presents various challenges. Examples are the prevention of the rapid onset of muscle fatigue, the design of the co-actuation scheme with the other actuators (electric motor drives), and the selection of the appropriate stimulation waveforms and the duration of the maximum stimulation current during gait. The effector redundancy produced in a hybrid actuation (FES and electric motors) complicates the system and makes it hard to control.

There have been different proposals in literature to overcome the aforementioned problems. To address actuator redundancy problems and the rapid onset of muscle fatigue, Ha et al. (2016) presented two control loops to assist the hybrid actuation so as to optimize the issue of the onset of muscle fatigue. The first is based on tracking the desired joint trajectories, and the second uses joint torque profiles already available from previous steps to improve the motor torque efficiency, by shaping the muscle stimulation profile for the subsequent step. These two control loops provide feedback on the joint torque produced by the motor and FES so as to minimize the motor torque contribution required for a joint angle trajectory. Although the work presents a real-time control solution, the applied FES is not based on muscle dynamics but is designed to track some desired kinematics. Also, while fatigue is measured indirectly based on variations of motor current, it is considered to be generalized, i.e., there is no control of which muscle is the earliest to fatigue.

In the same line, Kirsch et al. (2016) established a switching control strategy to change between combined actuation and electromechanical actuation only in accordance with the subject's previously calibrated fatigue state. The main limitation of this work is that actuation is switched between FES and electromechanical actuators, i.e., when muscle fatigue is detected due to electrical stimulation, the controller disconnects the FES actuation and connects motor actuators to facilitate muscle recovery. When the recovery time is up, this motor actuation is disconnected, and the FES actuator is again applied to the subject.

To overcome the electromechanical delay when applying FES and the change in muscle performance over time, Del-Ama et al. (2014) proposed a controller to balance muscle and robotic actuation during walking. The main limitation is that the FES profiles are those that maintain the kinematics, and, as is also the case with the aforementioned works, the controller's efficiency must be further investigated with regard to therapeutic applications.

Regarding the design of the FES profiles, Sharma et al. (2014) proposed a dynamic optimization approach to compute the stimulation profiles. Using a biomechanical model, a customized range of stimulations can also be obtained to determine the optimal step length and walking speed. Nevertheless, the model is so complex that in practice it requires several simplifications to reduce the computational cost. Furthermore, Anderson and Pandy (2001b) had already previously demonstrated that dynamic and static optimization solutions were practically the same, and therefore that simulations can be optimized. Ferrante et al. (2016) proposed a method to design a personalized multi-channel FES controller for gait training based on muscle synergies, but electromechanical actuation is not considered. Doll et al. (2018) proposed an off-line dynamic optimization method to determine the minimum number of pulses that would maintain a constant desired isometric contraction force. The main drawback of the method is that only isometric contractions are considered, not muscle behavior during dynamic contractions. Also, only knee extensor muscles are considered. Alibeji et al. (2018) presented a controller in which dynamic postural synergies between the electric motors and FES of the muscles were artificially generated by means of optimizations. The main limitations of their study were the electromechanical delay, muscle fatigue, and actuator dynamics.

There is still no clear strategy to overcome early fatigue due to FES actuation. As mentioned above, some authors argue for switching between FES and electromechanical actuation based on the subject's specific fatigue state (Del-Ama et al., 2014; Ha et al., 2016; Kirsch et al., 2016). Others propose a leading actuation of the motors to ensure the appropriate kinematics and a pre-configured low-amplitude stimulation to improve rehabilitation therapy (Obinata et al., 2007; Farris et al., 2009; Kobetic et al., 2009). While this ensures kinematic guidance, the stimulation may not be enough to produce functional contractions of muscle tissue, and therefore the rehabilitation process may be compromised. To the best of our knowledge, no subject-specific design of stimulation profiles and the exoskeleton's electromechanical actuation has been proposed in the literature. Došen and Milovanović (2009) proposed a form of dynamic optimization to obtain FES profiles with which to track some desired kinematics, but the exoskeleton was not included.

The aforementioned works provide a methodological approach to controlling the hybrid exoskeleton so as to follow some desired kinematics. Nevertheless, the FES profiles applied are those that track the proposed kinematics, not the kinematics expected in a physiological contraction. Consequently, the rehabilitation process may lack neurophysiological feedback. The objective of the present work was twofold: first, to develop a method to simultaneously calculating the FES profiles and the electromechanical actuation of hybrid orthosis; and second, to design orthosis actuation and FES profiles that consider fatigue.

## 2. Methods

**Figure 1:**
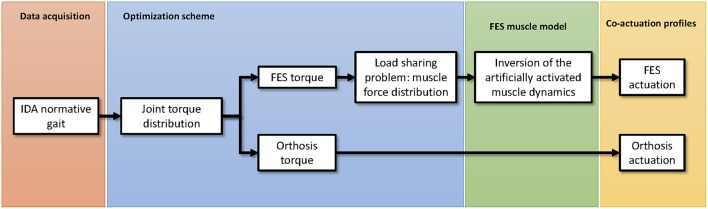
Schematic representation of the proposed algorithm to estimate the co-actuation profiles in hybrid orthoses.

The proposed algorithm to estimate the combined actuation of both the electromechanical and the FES actuators is presented in Figure 1. Briefly, the process starts with the inverse dynamics analysis of a normative gait. Alternatively, the normative motion can be obtained from gait databases (see e.g., Liu et al., 2008; Rajagopal et al., 2016). Once the net joint torques and joint reaction forces are known, the idea is to distribute them between the orthosis and FES systems. The orthosis torque profile can be directly applied in the electromechanical actuator. The FES torque, however, must be distributed among muscles. To do so, a load sharing problem must be solved. Once the distribution of forces among the muscles spanning any of the selected joints has been obtained, the artificially activated muscle model can be inverted to finally obtain the stimulation profiles that have to be applied to the selected muscles to provide the given FES torque. The following subsections will describe the different stages of the algorithm proposed to obtain the actuation profiles for the hybrid exoskeleton. The description of the method is based on the scheme depicted in Figure 1, and therefore we first present the data acquisition of the normative gait, then the inverse dynamics analysis (IDA), followed by the approach to the load sharing problem in order to estimate the contribution of each actuator, and lastly the inversion of the artificially activated model.

### 2.1. Data Acquisition: Normative Gait

As one objective is to define the actuation profiles for the hybrid exoskeleton, we must first define the desired motion. Since the intended wearer of the exoskeleton is unable to perform normal motion, gait databases can be used (Liu et al., 2008; Rajagopal et al., 2016) and adapted to the anthropometric data of the specific subject. In this work, we used the database of Liu et al. (2008), and the model weighed 72.6 kg with no history of neurological disorders. To perform the inverse dynamics analysis, the latest version of OpenSim software was used (Delp et al., 2007; Seth et al., 2018) (OpenSim, RRID:SCR002683).

### 2.2. Biomechanical Model

The biomechanical model used has 23 degrees of freedom and 92 actuators. It consists of 12 rigid bodies, and the joints are representative of the allowed motion, i.e., revolute joints at the knee or ankle, spherical joints at the hip, etc. The motion of the model is restricted to the sagittal plane, thus reducing the actuators to 15 in each leg (see Table 1). The actuators correspond to the model's flexor and extensor muscles. The rigid bodies are characterized by their mass, length, moment of inertia about the center of mass, and distance from the center of mass to the proximal joint. The equations of motion can be written as:

(1) $$\{\begin{array}{c} M\ddot{q}+\Phi_{q}^{T}\lambda =Q\\ \Phi (q,t)=0 \end{array}$$

where **M** is the system's mass matrix, $\ddot{\text{q}}$ the acceleration vector, $\Phi_{\text{q}}^{T}\lambda$ the generalized forces associated with the Lagrange multipliers (λ), and **Q** the generalized force vector. Φ is the constraint equations vector, and Φ**_q_** is the Jacobian matrix of the constraint equations. The net joint reaction forces and net driver (human-orthosis actuation) moments during some physical activity or motion can be estimated using kinematic and anthropometric data in Equation (1) together with information given by the force plates.

**Table 1:** Classification of the muscles used in this work according to the joint (hip, knee, or ankle) and movement (flexion, extension) provided.

**Joint**	**Movement**	**Muscle**
Hip	Flexion	Rectus femoris
	Extension	Gluteus maximus (1,2,3)
		Adductor magnus
		Semitendinosus
		Semimembranosus
		Biceps femoris long head
		Biceps femoris short head
Knee	Flexion	Semitendinosus
		Semimembranosus
		Biceps femoris long head
		Biceps femoris short head
		Gastrocnemius medial
		Gastrocnemius lateral
	Extension	Rectus femoris
		Vastus lateralis
		Vastus medialis
Ankle	Dorsiflexion	Tibialis anterior
	Plantar Flexion	Gastrocnemius medial
		Gastrocnemius lateral
		Tibialis posterior

### 2.3. Co-actuation: Estimation of FES and Electromechanical Actuation Profiles

Since several muscles serve each joint of the skeletal system, muscle forces cannot be directly computed from joint moments. This is the well-known redundant actuator problem in biomechanics. In order to solve this problem, optimization procedures are used. Various methods (static optimization, dynamic optimization, augmented static optimization, large-scale static optimization) and criteria (minimum metabolic cost of transport, minimum sum of muscle stresses, minimum hyper-extension of the joints, time-integral cost of activations, torque-tracking) for this optimization are available in the literature (Crowninshield and Brand, 1981; Nigg and Herzog, 1999; Anderson and Pandy, 2001a,b; Menegaldo et al., 2006; Ambrosio and Kecskemethy, 2007; Pipeleers et al., 2007; Rengifo et al., 2010). See Ojeda (2012) for a review of the optimization methods, and Ou (2011) for a review of the optimization criteria. The optimization assumes that the load sharing among the muscles follows certain rules during learned motor activities, and that the muscle recruitment strategy is governed by physiological criteria aimed at achieving functional efficiency. In order to quantify the simultaneous muscle and active orthosis contributions to the net joint torques of the human-orthosis system, this analysis considered the aforementioned 15 muscle groups per leg and three external torques applied to the ankles, knees, and hips. The external actuation proposed in this theoretical approach is the complete case in the sense that it is an active hip-knee-ankle-foot orthosis (A-HKAFO) to provide hip, knee, and ankle joint moments that assist the pathological gait of impaired subjects. Furthermore, as many of the muscles considered are biarticular (spanning two rather than just one joint), the optimization problem should consider all the lower limbs joints simultaneously (Michaud et al., 2015). For an ankle-foot orthosis (AFO) or knee-ankle-foot orthosis (KAFO), the problem is solved in the same way but neglects the contribution of the electromechanical actuation at the hip and knee joints in the first case, and the hip in the second case.

### 2.4. Joint Torque Distribution

Inverse dynamics-based static optimization methods have been known for more than three decades. The net joint torques are calculated using the inverse dynamics approach, and then the muscle load sharing problem is solved at each time step by minimizing a cost function $J(F^{M})$ that depends on muscle forces (e.g., the sum of muscle stresses). This optimization problem is subject to two constraints: that the sum of muscle moments must equal the net joint torque obtained by inverse dynamics, and that the maximum possible muscle force is limited by their maximum isometric force, $F_{0}^{M}={\left[f_{0,1}^{M},\ldots ,f_{0,n}^{M}\right]}^{T}$ (Crowninshield and Brand, 1981). The results are that the muscle forces provide the acquired motion. However, for impaired subjects or for cases in which the motion is provided by the exoskeleton, the net joint torque must also be distributed between the electromechanical actuator and the natural actuators (i.e., the muscles). The formal expression of this problem is given by Alonso et al. (2012):

(2) $$\begin{array}{l} min\;J(F^{M},T_{o})\\ s.t.\;R\;\cdot \;F=T\\ \;0\leq F^{M}\leq F_{0}^{M}\\ \;-T_{o}^{*}\leq T_{o}\leq T_{o}^{*} \end{array}$$

where J is a cost function that depends on the muscle (**F^M^**) and orthosis actuation (**T_o_**) vectors. In particular, $F={\left[F^{M},T_{o}\right]}^{T}={\left[f_{1}^{M},\ldots ,f_{n}^{M},T_{o,1},\ldots ,T_{o,m}\right]}^{T}$ is the muscle and orthosis actuation vector at each instant, *n* the number of muscle groups, *m* the number of joint actuators, **R** the matrix of equivalent moment arms of the different muscle groups and orthosis actuators, **T** the vector of net joint torques obtained from IDA, and $F_{0}^{M}={\left[f_{0,1}^{M},\ldots ,f_{0,N}^{M}\right]}^{T}$ the vector of maximum isometric forces that limits the maximum possible muscle actuation. Moment arms are defined as the distance between the muscle's line of action and the joint's axis of rotation. The muscle lengths and moment arms can be obtained from the OpenSim IDA results. The moment arms of each muscle with respect to ankle (*r*_*a*_), knee (*r*_*k*_), and hip (*r*_*h*_) are considered to be variables of the motion. The orthosis actuation moment arm is taken to be 1 for the actuated joint and 0 for the rest. The third constraint above is to ensure that the orthosis actuation does not exceed the maximum available torque.

**Figure 2:**
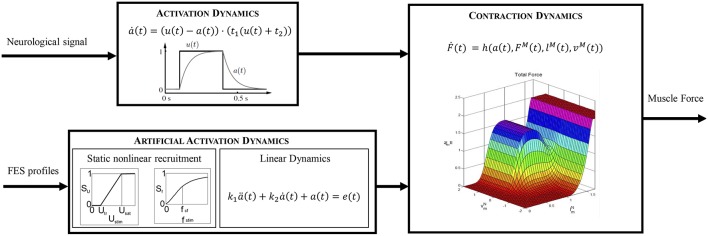
Comparison of the different dynamics that lead to muscle force production.

Static optimization (SO) is computationally more efficient than dynamic optimization since it does not require multiple integrations of the equations of motion. Nevertheless, it does not consider the activation and contraction dynamics of the muscle (see Figure 2, top), which can lead to physiologically inconsistent results. In the present work we therefore use the so-called physiological static optimization (PSO) approach (Alonso et al., 2012) which is a modification of the classical SO approach that considers muscle physiology. This scheme maintains the computational efficiency relative to dynamic optimization approaches while considering the muscle contraction dynamics, thus ensuring the physiological consistency of the solution obtained. The approach consists of two steps. In the first, the inverse contraction dynamics problem is solved, assuming that muscle activations are maximal at every instant, i.e., $\text{A}={\left[a_{1},\ldots ,a_{N}\right]}^{T}={\left[1,\ldots ,1\right]}^{T}$, where **A** is the activation vector and *a*_*j*_ are the activation values for each muscle (*j* = 1, …, *N* = 15). In particular, the contraction dynamics ordinary differential equation

(3) $${\dot{f}}^{M}(t)=g(a(t),f^{M}(t),l^{M}(t),{\dot{l}}^{M}(t))$$

is integrated given *a* = 1 for all muscles once the values of *l*^*M*^(*t*) and ${\dot{l}}^{M}\left(t\right)$ are known from the generalized coordinates of the multi-body model, i.e., from the OpenSim IDA results. The resulting muscle forces, expressed as *f*^*M*, *^(*t*) are the maximum achievable muscle forces compatible with the measured kinematics considering full activation of muscle tissue. For the sake of simplicity, the tendon is considered to be stiff (a rigid element). The result is the trajectory of the maximally achievable muscle force for each muscle.

In the second step, these force vectors are scaled to the real activations by solving an optimization scheme. Specifically, the activations compatible with the net joint torques obtained by inverse dynamics are calculated using a static optimization approach. The design variables are the activation vector, **A**, and the orthosis actuation vector, **T_o_**. The cost function F is the sum of a function of muscle contribution to the net joint torque, $J\left({\text{A}}_{m}\right)$, and a function of the electromechanical actuation, $H\left({\text{T}}_{o}\right)$:

(4) $$\begin{array}{l} \;Min\;F=J(A)+H(T_{o})\\ subject\;to\;R\;\cdot \;\left(A_{m}\;\cdot \;F^{*}\right)=T,\\ \;J(A)\;\cdot \;H(T_{o})\leq 0\\ \;0\leq a_{j}\leq 1,\;j=1,\ldots ,N=15,\\ \;T_{min}\leq T_{o,k}\leq T_{max},\;k=1,2,3 \end{array}$$

where $F^{*}={\left[F^{M,*},T_{o}^{*}\right]}^{T}={\left[f_{1}^{M,*},\ldots ,f_{n}^{M,*},T_{o,1}^{*},\ldots T_{o,k}^{*}\right]}^{T}$ is the maximum muscle and orthosis actuation vector at each instant, $A_{m}={\left[A,T_{o}\right]}^{T}={\left[a_{1},\ldots ,a_{n},T_{o,1},\ldots T_{o,k}\right]}^{T}$ is the design variable vector with *a*_*j*_ being the activations for each muscle and *T*_*o,k*_ the orthosis actuations at each joint. The first (equality) constraint ensures that the contribution of the two actuation profiles equals the net available torque, **T**. The second (nonlinear inequality) constraint ensures that the muscle and orthosis torques always assist each other. The third constraint bounds the values of the activation to between 0 and 1. And the fourth is used to allow both flexion and extension for the electromechanical actuation within the bounds set by the torque limits.

The cost function F may be expressed in different forms depending on the physiological criteria selected (Rasmussen et al., 2001; Ou, 2011). It has a strong influence on the result (Michaud et al., 2015). The term corresponding to the electromechanical actuation implies that one has to use either dimensionless cost functions, or the same units for both the electromechanical and the muscular actuations (provided by FES). A general expression for the cost function in the optimization problem can be written as:

(5) $$\begin{array}{l} F=J(A)+H(T_{o})=\delta \;\cdot \;\sum_{j=1}^{N_{m}}{\left(h_{1}\left(a_{j}(t),f_{j}^{M}(t)\right)\right)}^{n}\\ \;+\left(1-\delta \right)\;\cdot \;\sum_{k=1}^{N_{j}}{\left(h_{2}\left(T_{o,k}(t)\right)\right)}^{n} \end{array}$$

where *h*_1_ and *h*_2_ are different functions (see the list below) and *N*_*m*_ and *N*_*j*_ are the numbers of muscles and joints, respectively. We tested four cost functions.

- CF1: Force and torque normalized to the *j* − *th* isometric muscle force ($f_{0,j}^{M}$) and the *k* − *th* maximum torque ($T_{o,k}^{max}$):
  (6) $$F_{1}=\delta \cdot \sum_{j=1}^{N}{\left(a_{j}(t)\cdot \frac{f_{j}^{M,*}(t)}{f_{0,j}^{M}}\right)}^{n}+\left(1-\delta \right)\cdot \sum_{k=1}^{3}{\left(T_{o,k}(t)\cdot \frac{1}{T_{o,k}^{max}}\right)}^{n}$$
- CF2: Muscle and electromechanical power:
  (7) $$\begin{array}{l} F_{2}=\delta \cdot \sum_{j=1}^{N}{\left(-a_{j}(t)\cdot f_{j}^{M,*}(t)\cdot v_{j}^{M}(t)\right)}^{n}\\ \;+\left(1-\delta \right)\cdot \sum_{k=1}^{3}{\left(T_{o,k}(t)\cdot {\dot{\theta }}_{k}(t)\right)}^{n} \end{array}$$
- CF3: Force and torque normalized to the *j*−*th* maximum value of the trajectory of maximally achievable muscle force ($f_{j}^{max}$) in the cycle and the *k*−*th* maximum torque ($T_{o,k}^{max}$), respectively:
  (8) $$F_{3}=\delta \cdot \sum_{j=1}^{N}{\left(a_{j}(t)\cdot \frac{f_{j}^{M}(t)}{f_{j}^{max}}\right)}^{n}+\left(1-\delta \right)\cdot \sum_{k=1}^{3}{\left(T_{o,k}(t)\cdot \frac{1}{T_{o,k}^{max}}\right)}^{n}$$
- CF4: Largest relative muscle force and torque normalized to the *k*−*th* maximum torque ($T_{o,k}^{max}$).
  (9) $$\begin{array}{l} F_{4}=max\{a_{1}(t)\cdot \frac{f_{1}^{M,*}(t)}{f_{1}^{max}},\ldots ,a_{N}(t)\cdot \frac{f_{N_{m}}^{M,*}(t)}{f_{N_{m}}^{max}},\frac{T_{o,1}(t)}{T_{o,1}^{max}},\\ \;\ldots ,\frac{T_{o,N_{j}}(t)}{T_{o,N_{j}}^{max}}\} \end{array}$$

where *n* is usually set to 2 (Michaud et al., 2015), although different values have been applied in the literature Ou (2011). According to Yamaguchi (2005), contraction velocity *v*^*M*^(*t*) can be expressed as $v^{M}\left(t\right)=-{\dot{l}}^{M}\left(t\right)$ in Equation (7). Rasmussen et al. (2001) proposed a method to deal with a min/max objective function ($F_{4}$). Their procedure generates activation patterns consistent with contraction dynamics only if muscle force (*f*^*M*^) scales linearly with muscle activation. Although this is certainly not the case for standard Hill models, this assumption has been widely used in the literature, and is the basis of OpenSim's static optimization algorithm. In this first approach, we take the weighting factors to be the same for both actuators (δ = 0.5). The weighting factors can be associated with the level of assistance in a hybrid orthosis (Anaya et al., 2018). The parameter δ basically represents some priority given to the use of either the FES actuation or the motor actuators. A higher value of δ prioritizes muscle actuation through FES, and a lower value reduces FES actuations and increases the torque provided by the electromechanical actuation. Parameters accounting for atrophy should be considered in the computational model if present in the subject under analysis. For instance, various studies (Amankwah et al., 2004; McDonald et al., 2005) have shown that passive torque tends to be greater in pathological participants than in healthy ones, especially in the ankle and hip joints.

At the end of this step, two signals are available: the electromechanical actuation joint torque which can be applied directly to the exoskeleton, and the activation signal for each muscle that scales the maximum muscle force temporal histories. In the following subsection, these activation signals will be used as inputs in the artificial activation dynamics to calculate the FES profiles to apply to the subject so as to obtain the joint torque calculated in this step.

### 2.5. Estimation of FES Profiles

The dynamic behavior of a muscle is modeled by means of two cascaded differential equations (Zajac, 1989): the excitation-to-activation dynamics which describes the transformation of a neural signal into muscle recruitment levels, and the activation-to-force dynamics which represents the transformation of an activation signal into muscle force (Figure 2, top). For an artificially stimulated muscle (Figure 2, bottom), the contraction process is considered to be the same as in the physiological case since the muscle parameters considered in the contraction dynamics do not vary significantly. Nevertheless, the excitation-to-activation dynamics do change, since FES artificially induces a current in specific motor neurons, not in muscle tissue (Lynch and Popovic, 2008).

#### 2.5.1. Excitation-to-Activation Dynamics in Physiologically Activated Muscles

According to Nagano and Gerritsen (2001), the excitation-to-activation dynamics of a healthy, physiologically activated muscle (Figure 2, top) are described by:

(10) $$\dot{a}(t)=(u(t)-a(t))\cdot (t_{1}u(t)+t_{2})$$

where *a*(*t*) is the muscle activation, *u*(*t*) the excitation signal, and *t*_2_ = 1/*T*_*fall*_ and *t*_1_ = 1/(*T*_*rise*_ − *t*_2_) parameters depending on time constants *T*_*rise*_ and *T*_*fall*_ (Nagano and Gerritsen, 2001). This equation transforms an idealized muscle excitation signal, a dimensionless value between 0 and 1, into delayed muscle activation levels, also constrained to the same range of values.

#### 2.5.2. Excitation-to-Activation Dynamics in FES-Activated Muscles

In the case of a subject with gait disability (spinal cord injury, post-polio syndrome, knee extensor failure or weakness, etc.), the natural path of the neural signal to the muscles is interrupted in some way. It has been proven that the use of FES to induce muscle contractions under these circumstances has some benefits for the patient. The activation signal produced by FES depends on the stimulus's intensity and frequency, where the former can be controlled by the amplitude or pulse width of the stimulus signal. In the literature, there are mathematical models that describe excitation-to-activation dynamics in FES-induced contractions: Makssoud et al. (2004) presented an FES muscle model based on Huxley's cross-bridge theory, which was divided into activation and contraction parts, with the former accounting for stimulation intensity, pulse width, and frequency. Watanabe et al. (1999) presented a mathematical description of the frequency-force relationship which was completed by Gföhler et al. (2004) by including the effects of amplitude. In the present work, this last model is used to obtain an estimate of FES profiles in terms of intensity or of pulse width and/or frequency. The activation dynamics for this type of induced contraction can be represented by a nonlinear static block (related to stimulus frequency and intensity) coupled with a linear dynamics block represented by a second-order differential equation (relating FES and activation signals) by means of a two-block Hammerstein structure (Durfee and McLean, 1989).

The excitation signal, *e*(*t*), output of the first block, combines the influence of stimulus intensity, *U*_*stim*_ (in terms of amplitude or pulse width) and frequency, *f*_*stim*_, and can be expressed as:

(11) $$e(t)=S_{u}\cdot S_{f}$$

where *S*_*u*_ and *S*_*f*_ are scaling factors for stimulus intensity and frequency, respectively. The first factor corresponds to an isometric recruitment curve divided into three regions. In the first, no muscle fibers are recruited below a threshold (*U*_*tr*_); in the third, all fibers are recruited above the saturation level (*U*_*sat*_); and, in the intermediate region, there is active recruitment between those limits (Gföhler et al., 2004):

(12) $$S_{u}=\{\begin{array}{ccc} 0 & for & U_{stim}<U_{tr}\\ \frac{U_{stim}-U_{tr}}{U_{sat}-U_{tr}} & for & U_{tr}\leq U_{stim}\leq U_{sat}\\ 1 & for & U_{stim}>U_{sat} \end{array}$$

As this expression represents a process of scaling between intensity threshold and saturation levels, either a pulse-width or an amplitude signal can be used as the input *U*_*stim*_. The threshold and saturation values can be measured experimentally. The former corresponds to the amplitude of the input signal that produces the first effective contraction (minimal variation in the joint angle). The latter is the value of the amplitude beyond which no more motion is observed during muscle contraction. Both values depend strongly on the subject's morphology, muscle atrophy, treatment with botulinum toxin, and sensitivity, and they must be measured independently for each muscle. The values of *U*_*stim*_ may range from 10 to 50 mA. Greater values combined with higher stimulation frequencies or different pad sizes may cause skin burns or neuromuscular injuries (Martín, 2004). In the present work, we have assumed equal physiological actuators since we have no access to the database's subjects to perform any measurements. This assumption also maintains the simplicity of the calculations.

The second factor in Equation (11) has been defined as (Watanabe et al., 1999):

(13) $$S_{f}=\frac{k_{1}-k_{2}}{1+e^{(f_{stim}-f_{0})/R}}+k_{2}$$

where *k*_1_, *k*_2_, *R*, and *f*_0_ are appropriate constants. The values of *a*_1_ and *f*_0_ can be obtained by assuming *S*_*f*_ = 0 at *f* = 0, and *S*_*f*_ = 1 at the critical fusion frequency (*f* = *f*_*CF*_):

(14) $$k_{1}=-k_{2}e^{-f_{0}/R}$$

(15) $$f_{0}=R\;\cdot \;ln\left[(k_{2}-1)\cdot e^{f_{CF}/R}-k_{2}\right]$$

where *k*_2_ is the ratio of the maximum force to the force at *f*_*CF*_, i.e., *k*_2_ = *F*_*max*_/*F*_*CF*_, and can be determined experimentally. In the present work, we set the parameter *R* to 15, although it can also be measured on patients (Watanabe et al., 1999).

The second block of the Hammerstein structure can be represented as a second-order ordinary differential equation (Gföhler et al., 2004):

(16) $$\begin{array}{r} k_{1}\cdot ä\left(t\right)+k_{2}\cdot ȧ\left(t\right)+a\left(t\right)=e\left(t\right) \end{array}$$

where *k*_1_ = *T*_*e*_ · *T*_*rise*/*fall*_ and *k*_2_ = (*T*_*rise*/*fall*_ + *T*_*e*_), with *T*_*rise*_ and *T*_*fall*_ being time constants (Nagano and Gerritsen, 2001), and *T*_*e*_ a time constant for the excitation of artificially stimulated muscles. These constants depend on the physiological cross-section area, muscle mass, and fast-twitch muscle fiber percentage (Gföhler et al., 2004). The model therefore takes into consideration atrophy in disabled patients, which is usually associated with those values.

As the activations of each muscle are known from the previous step, the excitation signal *e*(*t*) can be obtained directly from Equation (16) using backward differences and interpolating the last values with splines to avoid the loss of values during the process. If the stimulus frequency is fixed at typical values, i.e., 20–40 Hz, it is then possible to calculate *S*_*f*_ from Equation (13) and then *S*_*u*_ from Equation (11) to solve *U*_*stim*_ in Equation (12), and thus obtain the stimulation profile in terms of variable amplitude and constant frequency. Contrariwise, if the stimulus amplitude is fixed between typical values of 20–35 mA, then it is possible to calculate *S*_*u*_ from Equation (12), then *S*_*f*_ from Equation (11), and last *f*_*stim*_ from Equation (13) to obtain the stimulation profile in terms of variable frequency and constant amplitude.

### 2.6. Fatigue in FES-Induced Contractions

One of the major drawbacks when dealing with artificial activation of muscle tissue is the lack of selectivity in muscle fibers. A characteristic tetanic contraction that produces movement in physiologically activated muscles is defined by the sequential stimulation of adjacent fibers at a frequency of 6-8 Hz. This sequential recruitment guarantees a value of fatigue in accordance with the activity. Contrariwise, in a FES-induced contraction, in which the system is stimulated at 20–40 Hz, the individual motor units are not stimulated sequentially. Instead, all types of fibers (type I, slow; type IIa, mid; and type IIb, fast) are stimulated at the same time with the consequent early onset of fatigue, since type IIb (fast) fibers present high levels of force production but also have poor fatigue resistance (Lynch and Popovic, 2008; Vromans and Faghri, 2018).

Since a hybrid exoskeleton must facilitate motion, fatigue effects should also be considered. In order to maintain the level of actuation, clinicians often increase stimulation intensity or frequency. Unfortunately, an increase of either of these parameters accelerates the onset of fatigue (Ding et al., 2003). This may be counter-productive during FES training. When using an exoskeleton, however, a variation of those parameters may contribute to prolonging the electromechanical actuation battery life.

There are several studies in the literature that address mathematical models of muscle fatigue under FES (Ding et al., 2003; Cai et al., 2010; Marion et al., 2013). They are mainly based on the physiological mechanism. These models are complicated to use in an IDA approach because of their large number of variables. In order to evaluate the evolution of the combined FES actuation and electromechanical actuation, we hold to the idea of calculating the co-actuation with the inclusion of a fatigue term. Tepavac and Schwirtlich (1997) proposed an exponential decay to describe the reduction in muscle force production under FES in the first 5 min of electrical stimulation. Chou and Binder-Macleod (2007) measured a 50% reduction in the peak force in the first 180 s. These variations must be considered when designing appropriate actuation profiles.

From this approach, there arises a new fatigue cost function (G) which accounts for the decrease in muscle actuation due to FES-induced fatigue:

(17) $$\begin{array}{r} G=J\left(\text{A}\right)+H\left({\text{T}}_{o}\right)=\delta \cdot \overset{N}{\underset{j=1}{\sum }}h_{1}\left(a_{j},f_{j}^{M}\right)\cdot \psi_{p}\left(t\right)+\left(1-\delta \right)\cdot \overset{3}{\underset{k=1}{\sum }}h_{2}\left(T_{o,k}\right) \end{array}$$

where *h*_1_ and *h*_2_ are the functions described in Equations (6–9), and ψ_*p*_(*t*) represents a fatigue function that limits the actuation, where *p* describes the type of the function used (see Equations 18 and 19). We shall compare two different fatigue functions. The first represents an exponential decay that models the peak force reduction observed in the aforementioned works. It can be expressed as:

(18) $$\begin{array}{r} \psi_{exp}\left(t\right)=e^{-C_{1}\cdot t-C_{2}}+C_{3} \end{array}$$

where *C*_1_, *C*_2_, and *C*_3_ are appropriate constants to model a decay of some 80% in peak force with a gentle slope. For the present work, we set these values to *C*_1_ = 0.02, *C*_2_ = 0.3, and *C*_3_ = 0.2. The actuation profiles resulting from the optimization already consider muscle fatigue, and therefore, to compensate for the variation in muscle actuation, variation in electromechanical actuation is also considered.

The second fatigue function is that proposed by Riener et al. (1996):

(19) $$\begin{array}{r} \frac{d\psi_{R}\left(t\right)}{dt}=\frac{\left(\psi_{R}^{min}-\psi_{R}\left(t\right)\right)\cdot a\left(t\right)}{T_{fat}}+\frac{\left(1-\psi_{R}\left(t\right)\right)\cdot \left(1-a\left(t\right)\right)}{T_{rec}} \end{array}$$

where ψ^*min*^ is the minimum value achievable when a muscle is fatigued, and *T*_*fat*_ and *T*_*rec*_ are time constants representing fatigue and recovery times. In this work, we took the values proposed by Riener et al. (1996) for these constants. The use of these dynamics requires a slight modification in the optimization routine. At each step, the required activations for cooperative actuation are calculated by means of the proposed algorithm. These activations are then used to compute the fatigue function, which basically reduces muscle force capacity by scaling the activation. A second optimization is then computed to calculate simultaneously the muscle activations under fatigue conditions (and therefore FES profiles) and the compensated orthosis actuation profiles.

## 3. Results and Discussion

**Figure 3:**
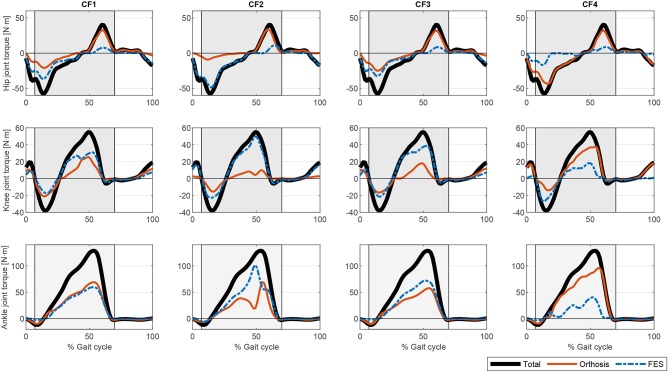
Comparison of the different torque profiles for the proposed optimization cost functions.

**Figure 4:**
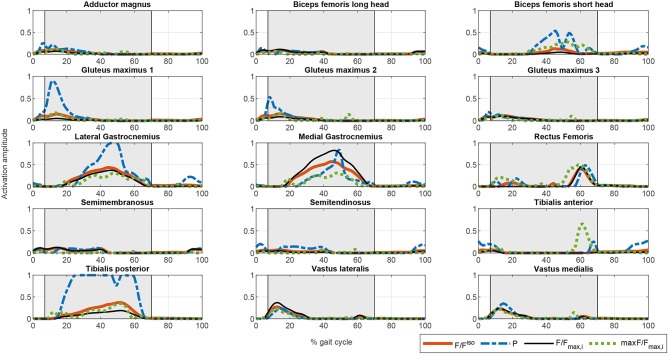
Comparison of the different activation profiles for the proposed optimization cost functions. CF1: Thick solid orange line. CF2: Dashed blue line. CF3: Thin solid black line. CF4: Dotted green line.

The procedure described was implemented in Matlab (The MathWorks Inc.) running on an Intel^(R)^ Core^(TM)^ i5 CPU 650 at 3.20 GHz. We used the Matlab *fmincon* routine for CF1 to CF3, and *fminimax* for CF4. Simulation times (as obtained for a single cycle) were 28.35 s for CF1, 8.83 s for CF2, 18.87 s for CF3, and 35.69 s for CF4. The different cost functions evaluated yielded different actuation profiles. Figure 3 shows the output of the optimization algorithm in terms of joint torque distribution. The “softest” results were obtained with cost functions CF1 and CF3. Normalizing the muscle force to the isometric muscle force $f_{0,j}^{M}$ (CF1) instead of to the maximum force in each cycle $f_{j}^{max}$ (CF3) led to lower calculated joint torques related to muscle actuation. Although the results for the hip seem to be similar, there is a greater muscle contribution in CF3 than in CF1, concordant with the activations shown in Figure 4. One observes that the contrary is the case for these cost functions in terms of orthosis actuation. In general, the use of muscle force-based cost functions (CF1, CF3, and CF4) results in similar contributions for the hip, but not for the other joints. The use of CF2 results in muscle actuators having greater relevance than electromechanical actuators. The differences in joint torque contribution for these two actuators may be explained by the individual contributions of the muscles to the joint torque.

The muscle activations for the different cost functions are depicted in Figure 4. As noted above, the results for CF1 and CF3 are similar but the muscle activation values for the latter are greater, reflecting the greater contribution of muscles to torque production. There are marked spikes in the CF2 activations, with, at some points, tetanic contractions that may be inappropriate for rehabilitation purposes or for smooth control of the degree of freedom with the two actuators.

**Figure 5:**
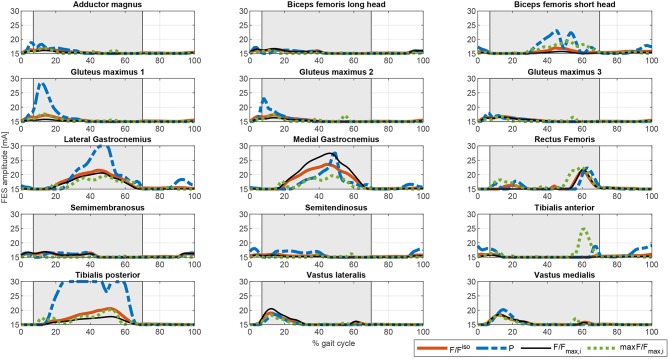
Comparison of the different FES profiles for the proposed optimization cost functions. CF1: Thick solid orange line. CF2: Dashed blue line. CF3: Thin solid black line. CF4: Dotted green line.

Once the artificial activation dynamics have been inverted, the FES profiles can be evaluated. For simplicity, we here assumed that all the muscles have the same threshold and saturation values. The results are shown in Figure 5. The process that leads from Figures 4, 5 (see section 2.5.2) is a temporal shift of the activation signal, followed by nonlinear scaling, and then normalization between the threshold and saturation levels. The main features of the activation profiles are preserved, i.e., the FES profiles obtained with CF2 are higher and at some points spiked, which, in terms of stimulation, may cause muscle tissue contractions that are hard to control. Moreover, a sustained FES-induced tetanic contraction, as in *tibialis posterior* or *lateral gastrocnemius*, may also result in early muscle fatigue.

**Figure 6:**
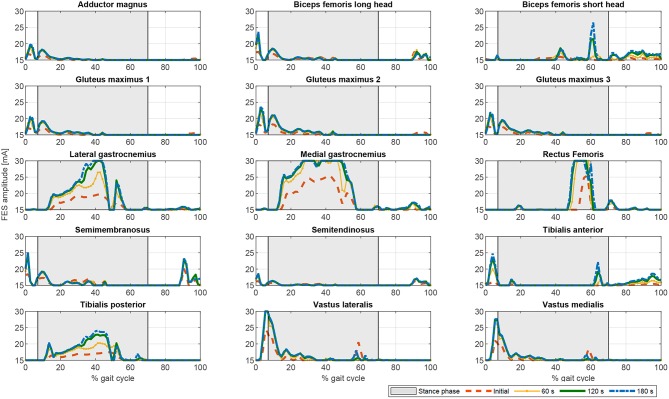
Evolution of the FES profiles for the proposed muscles using exponential decay fatigue factor for function $F_{1}$. Dashed orange line: Initial FES profile. Dotted yellow line: FES profile at 60 s. Solid green line: FES profile at 120 s. Dash-dotted blue line: FES profile at 180 s.

**Figure 7:**
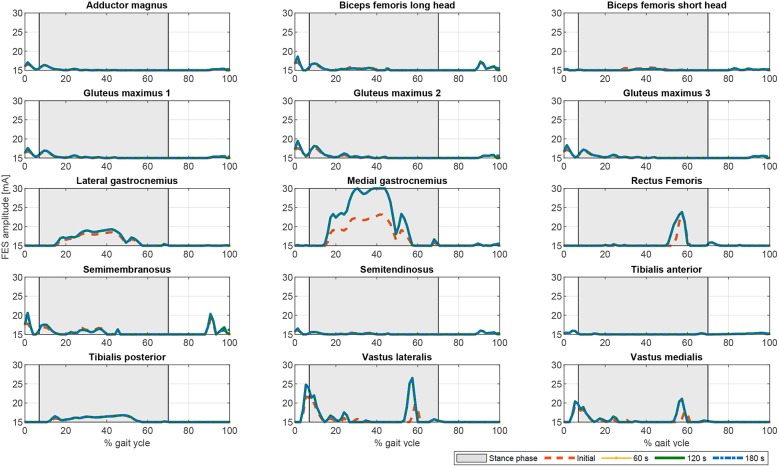
Evolution of the FES profiles for the proposed muscles using the fatigue factor proposed by Riener et al. (1996) for function $F_{1}$. Dashed orange line: Initial FES profile. Dotted yellow line: FES profile at 60 s. Solid green line: FES profile at 120 s. Dash-dotted blue line: FES profile at 180 s.

If fatigue is included in the optimization process by using Equation (17), the FES and electromechanical actuation time profiles are expected to vary during time. Both fatigue functions proposed in Equations (18) and (19) decrease exponentially being the first one softer than the second one. Another difference is that second one allows the muscle to recover partially. As the activations in the cost function are limited by this fatigue factor, which is decreasing over time, the results of the optimization process lead to increasing values of the activation profiles, and therefore of the FES profiles (see Figures 6, 7). As fatigue is compensated with increased values of muscle stimulation, the results in terms of joint torque are the same as in Figure 3 for CF1, i.e., the torque profiles remain constant and distributed as in Figure 3 for CF1 throughout the 180 s. These results are consistent with those of Del-Ama et al. (2014), in which the stimulation parameters are increased when fatigue appears to maintain a constant joint torque.

**Figure 8:**
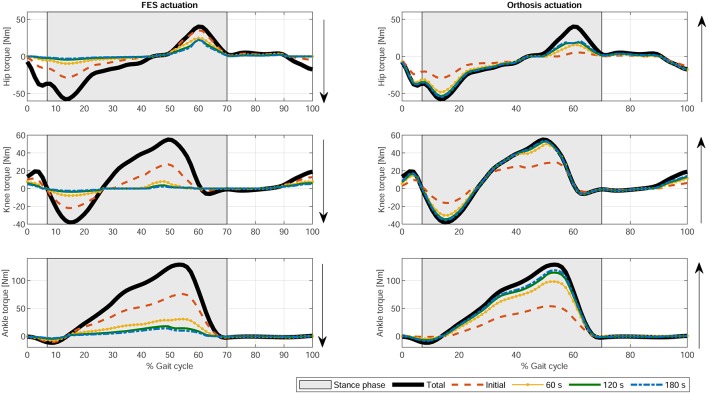
Evolution of the electromechanical and FES actuation at the hip, knee, and ankle joints using exponential decay fatigue factor for function $F_{1}$. Thick solid black line: Total joint torque. Dashed orange line: Initial joint torque. Dotted yellow line: Joint torque at 60 s. Solid green line: Joint torque at 120 s. Dash-dotted blue line: Joint torque at 180 s.

**Figure 9:**
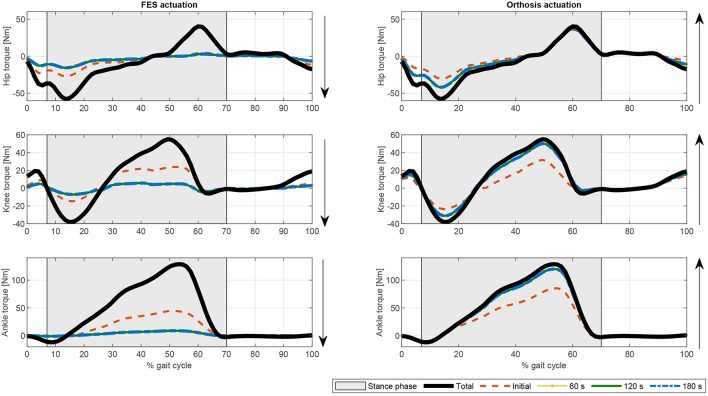
Evolution of the electromechanical and FES actuation at the hip, knee, and ankle joints the fatigue factor proposed by Riener et al. (1996) for function $F_{1}$. Thick solid black line: Total joint torque. Dashed orange line: Initial joint torque. Dotted yellow line: Joint torque at 60 s. Solid green line: Joint torque at 120 s. Dash-dotted blue line: joint torque at 180 s.

In terms of joint torque, if FES profiles of Figures 8, 9 are used, the contribution of FES and electromechanical actuation must be updated to reflect the effect of the fatigue factor. As the designed FES profiles increase over time, the contribution of the artificial contractions to joint torque decreases while the contribution of the electromechanical actuator must increase to compensate the effects of fatigue. These results are reflected in Figures 6, 7. Both show a decrease in FES actuation while the motor actuation increases. The exponential decay limits the actuation and, from the beginning, leads to a major but steady decline in amplitude that is sustained over the 180 s of the tested cycle. On the contrary, the dynamics proposed by Riener et al. (1996) presents a sharp decline in the first 50 s that must be compensated by the motor actuation. This strong decline in muscle force production may be due to the values of the time constants which were determined from patients with complete thoracic spinal cord injury. This factor reflects the characteristics of the two fatigue dynamics: in the first case, the exponential decay function is adapted in accordance with the observed reduction in muscle force production over 180 s, whereas, in the second case, although fatigue and recovery dynamics are considered, the muscle force reduction is constantly updated in accordance with the current state of the muscle, so that fatigue appears earlier in muscles with higher levels of stimulation, as in *medial gastrocnemius*, and the same level of stimulation can be applied and maintained in muscles with a lower stimulation profile, as in *adductor magnus* or *tibialis posterior*.

The present results are not directly comparable with those of previous work in which the proposed controller either switches between actuators and there is no information about the combined actuation, or there is only information for one actuator. Nevertheless, the results shown in Figure 3 are similar to those provided by Ha et al. (2016) for CF2 and CF3 at the knee level in which there was a reduction in the orthosis contribution to torque when FES was applied. Furthermore, the results obtained in this work explain, with a physiological model in the background, the use of bang-bang controllers to switch between orthosis and FES actuation when muscle fatigues or more complex controllers to switch between both actuators as in Ha et al. (2016) or Kirsch et al. (2016).

**Figure 10:**
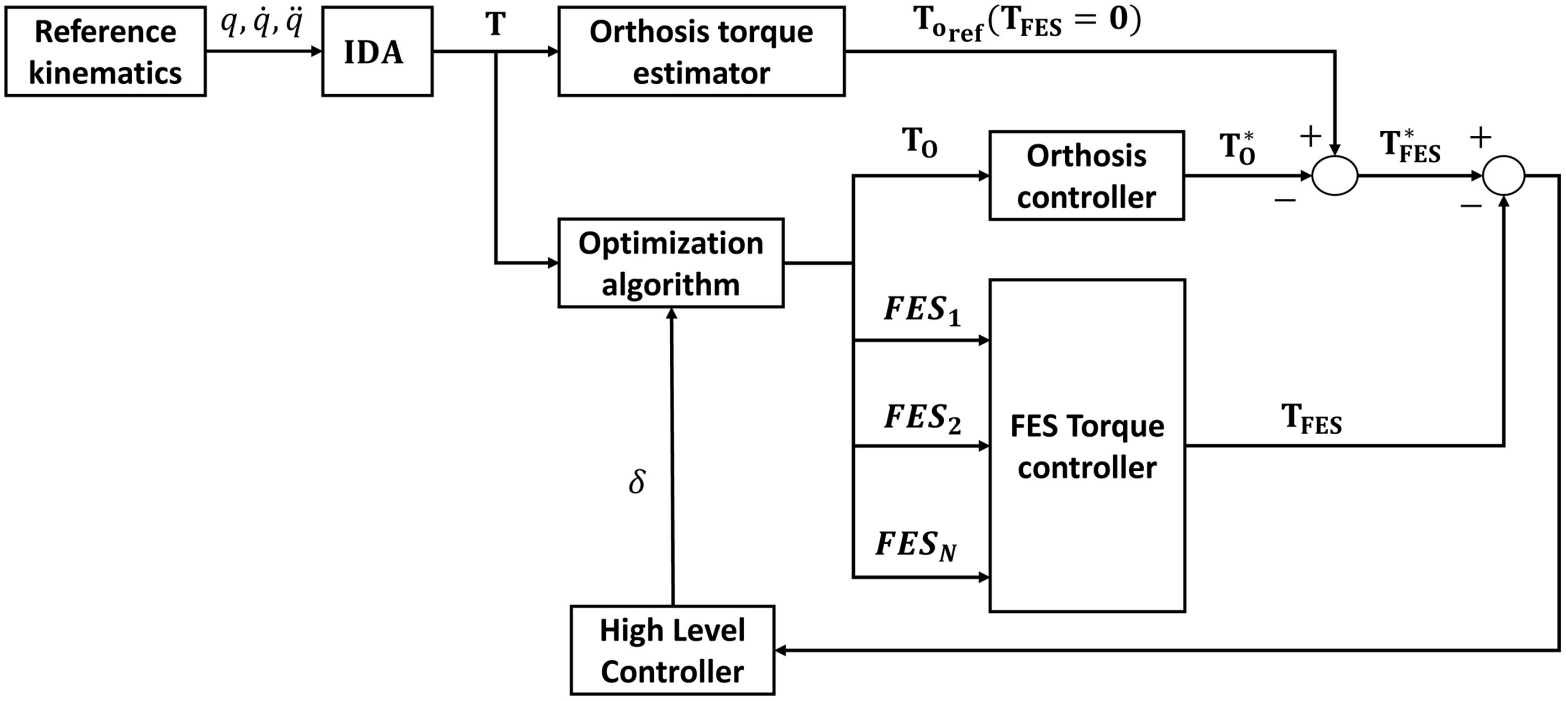
Control architecture of the cooperative controller.

The proposed method is off-line. Nevertheless, it could be applied to improve current control algorithms using the designed FES profiles which are physiologically consistent with the motion, instead of pre-defined ones that ensure kinematic guidance but may not have a rehabilitative function or result in delivering excessive electrical stimulation to the muscles causing either early fatigue or an exaggerated gait pattern (Anaya et al., 2018). For instance, Ha et al. (2016), Del-Ama et al. (2014), or Kirsch et al. (2016) do not design specific FES profiles for each muscle. In some cases, the FES profiles are already pre-defined, or the control algorithm switches between actuators to prevent fatigue. The method proposed here could be used to improve existing control algorithms, as in Ha et al. (2016), by including stimulation profiles that are physiologically consistent with the motion. Furthermore, according to Pizzolato et al. (2017), it might be possible to perform the inverse kinematics in real-time. By optimizing the programmed routines, cooperative control could be reached that is near real-time, or at most one step back, as in Ha et al. (2016), which may be enough for a cooperative controller that uses rehabilitative stimulation profiles. A possible solution for the control architecture of the cooperative controller is shown in Figure 10. A reference orthosis torque can be averaged (*T*_*o, ref*_) from the reference kinematics and total torque, measured in the absence of muscle stimulation. Then the proposed method would be used to predict a distribution of the required net joint moments between the motors and the artificially activated (i.e., electrically stimulated) muscles to comply with the desired kinematics. The difference between the reference and the predicted orthosis torques provides an estimate of the FES torque ($T_{FES}^{*}$). Then the difference between this value and the one predicted in the proposed scheme is used by a high-level controller to adapt the weightings (δ) in the optimization to reduce muscle fatigue. This scheme is similar to that proposed by Ha et al. (2016) but introduces muscle fatigue into the cooperative control of the hybrid orthosis by including the artificially activated muscle dynamics.

## 4. Conclusions

This work has described a method for the simultaneous design of the actuation provided by the electrical stimulator and the electromechanical actuators during gait assisted hybrid exoskeletons. The scheme ensures the physiological consistency of the results and is computationally efficient. There has been previous work (Ferrante et al., 2016; Ha et al., 2016; Alibeji et al., 2018) proposing methods for the control of such exoskeletons, but nothing regarding the design of the two actuation profiles at the same time. The present approach provided promising results for the definition of rehabilitation routines for hybrid exoskeletons or their control strategies. Furthermore, since fatigue was included in the model, estimates can be made of the rest intervals needed to improve muscle tissue recovery times. Despite the promising nature of the results, the following topics must be addressed to work toward a generalized solution:

- The optimization problem should consider the masses and inertias of the different lower limb segments of the exoskeleton since they may modify the joint torques, as well as the contact forces between the exoskeleton and the human body.
- The parameters used in the contraction dynamics must be measured on each subject. Also, the muscle stimulation threshold and saturation levels must be measured independently for each of the exoskeleton wearer's muscles. This may be a problem for some muscles due to the size of the pads and the crosstalk between muscles.
- Related to the previous item, the optimization problem of quantifying the minimum number of muscles to stimulate so as to produce some functional movement needs to be investigated further. To this end, and to reduce the dimensionality of the problem, the use of muscle synergies should be explored. This might reduce not only the complexity of donning and doffing the exoskeleton, but also the overall energy requirements of the system.
- For online applications, the value of δ should be optimized. A time-varying weighting factor may improve the trade-off between FES and orthosis actuation so as to put back the onset of muscle fatigue.
- Although a physiological criterion is applied in the load sharing problem, the contractions induced by electrical stimulation are non-physiological. The model proposed by Gföhler et al. (2004) already considers that modification in the activation dynamics, but it does not consider fatigue, therefore, further investigation is required in this area.
- While in a physiological contraction muscle fiber recruitment depends on the percentage of fast fibers by way of time constants (see Nagano and Gerritsen, 2001), muscle fibers during FES-induced contractions are all recruited together. This could be resolved by using time-dependent values instead of time constants to characterize the fatigue process.
- The results must be compared with an IDA of the gait assisted hybrid exoskeleton, i.e., the results need to be validated with the performance of tests.

## Data Availability

The datasets analyzed for this study are available on request from the corresponding author.

## Author Contributions

All authors contributed to writing the manuscript and approved its final version for submission.

### Conflict of Interest Statement

The authors declare that the research was conducted in the absence of any commercial or financial relationships that could be construed as a potential conflict of interest.

## References

[B1] Alibeji N. A. Molazadeh V. Dicianno B. E. Sharma N. (2018). A control scheme that uses dynamic postural synergies to coordinate a hybrid walking neuroprosthesis: theory and experiments. Front. Neurosci. 12:159. 10.3389/fnins.2018.00159 29692699 PMC5902565

[B2] Aliman N. Ramli R. Haris S. M. (2017). Design and development of lower limb exoskeletons: a survey. Robot. Auton. Syst. 95, 102–116. 10.1016/j.robot.2017.05.013

[B3] Alonso J. Romero F. Pàmies-Vilà R. Lugrís U. Font-Llagunes J. M. (2012). A simple approach to estimate muscle forces and orthosis actuation in powered assisted walking of spinal cord-injured subjects. Multibody Syst. Dyn. 28, 109–124. 10.1007/s11044-011-9284-5

[B4] Amankwah K. Triolo R. J. Kirsch R. (2004). Effects of spinal cord injury on lower-limb passive joint moments revealed through a nonlinear viscoelastic model. J. Rehabil. Res. Dev. 41, 15–32. 10.1682/JRRD.2004.01.0015 15273894

[B5] Ambrosio J. Kecskemethy A. (2007). Multibody dynamics of biomechanical models for human motion via optimization. Multibody Dyn. 4, 245–272. 10.1007/978-1-4020-5684-0_12

[B6] Anaya F. Thangavel P. Yu H. (2018). Hybrid fes–robotic gait rehabilitation technologies: a review on mechanical design, actuation, and control strategies. Int. J. Intell. Robot. Appl. 2, 1–28. 10.1007/s41315-017-0042-6

[B7] Anderson F. Pandy M. (2001a). Dynamic optimization of human walking. J. Biomech. Eng. 123:381. 10.1115/1.1392310 11601721

[B8] Anderson F. Pandy M. (2001b). Static and dynamic optimization solutions for gait are practically equivalent. J. Biomech. 34, 153–161. 10.1016/S0021-9290(00)00155-X 11165278

[B9] Cai Z. Bai E.-w. Shields R. K. (2010). Fatigue and non-fatigue mathematical muscle models during functional electrical stimulation of paralyzed muscle. Biomed. Signal Process. Control 5, 87–93. 10.1016/j.bspc.2009.12.001 23667385 PMC3647619

[B10] Chou L.-W. Binder-Macleod S. A. (2007). The effects of stimulation frequency and fatigue on the force–intensity relationship for human skeletal muscle. Clin. Neurophysiol. 118, 1387–1396. 10.1016/j.clinph.2007.02.028 17466581 PMC1993846

[B11] Crowninshield R. Brand R. (1981). A physiologically based criterion of muscle force prediction in locomotion. J. Biomech. 14, 793–801. 10.1016/0021-9290(81)90035-X 7334039

[B12] Del-Ama A. J. Gil-Agudo Á. Pons J. L. Moreno J. C. (2014). Hybrid fes-robot cooperative control of ambulatory gait rehabilitation exoskeleton. J. Neuroeng. Rehabil. 11:27. 10.1186/1743-0003-11-27 24594302 PMC3995973

[B13] Deley G. Denuziller J. Casillas J.-M. Babault N. (2017). One year of training with fes has impressive beneficial effects in a 36-year-old woman with spinal cord injury. J. Spinal Cord Med. 40, 107–112. 10.1080/10790268.2015.1117192 26832125 PMC5376139

[B14] Delp S. L. Anderson F. C. Arnold A. S. Loan P. Habib A. John C. T.. (2007). Opensim: open-source software to create and analyze dynamic simulations of movement. IEEE Trans. Biomed. Eng. 54, 1940–1950. 10.1109/TBME.2007.901024 18018689

[B15] Ding J. Wexler A. S. Binder-Macleod S. A. (2003). Mathematical models for fatigue minimization during functional electrical stimulation. J. Electromyogr. Kinesiol. 13, 575–588. 10.1016/S1050-6411(03)00102-0 14573372

[B16] Doll B. D. Kirsch N. A. Bao X. Dicianno B. E. Sharma N. (2018). Dynamic optimization of stimulation frequency to reduce isometric muscle fatigue using a modified hill-huxley model. Muscle Nerve 57, 634–641. 10.1002/mus.25777 28833237 PMC5817016

[B17] Došen S. Milovanović I. (2009). Design of optimal profiles of electrical stimulation for restoring of the walking. J. Automat. Cont. 19, 13–18. 10.2298/JAC0901013D

[B18] Durfee W. McLean K. (1989). Methods for estimating isometric recruitment curves of electrically stimulated muscle. IEEE Trans. Biomed. Eng. 36, 654–667. 10.1109/10.32097 2744790

[B19] Ekelem A. Goldfarb M. (2018). Supplemental stimulation improves swing phase kinematics during exoskeleton assisted gait of SCI subjects with severe muscle spasticity. Front. Neurosci. 12:374. 10.3389/fnins.2018.00374 29910710 PMC5992413

[B20] Farris R. J. Quintero H. A. Withrow T. J. Goldfarb M. (2009). Design and simulation of a joint-coupled orthosis for regulating fes-aided gait, in 2009 IEEE International Conference on Robotics and Automation (Kobe: IEEE), 1916–1922.

[B21] Ferrante S. Chia Bejarano N. Ambrosini E. Nardone A. Turcato A. M. Monticone M.. (2016). A personalized multi-channel fes controller based on muscle synergies to support gait rehabilitation after stroke. Front. Neurosci. 10:425. 10.3389/fnins.2016.00425 27695397 PMC5025903

[B22] Gföhler M. Angeli T. Lugner P. (2004). Modeling of artificially activated muscle and application to FES cycling. J. Mech. Med. Biol. 4, 77–92. 10.1142/S0219519404000850

[B23] Ha K. H. Murray S. A. Goldfarb M. (2016). An approach for the cooperative control of fes with a powered exoskeleton during level walking for persons with paraplegia. IEEE Trans. Neural Syst. Rehabil. Eng. 24, 455–466. 10.1109/TNSRE.2015.2421052 25915961

[B24] Herr H. (2009). Exoskeletons and orthoses: classification, design challenges and future directions. J. Neuroeng. Rehabil. 6:21. 10.1186/1743-0003-6-21 19538735 PMC2708185

[B25] Jimenez-Fabian R. Verlinden O. (2012). Review of control algorithms for robotic ankle systems in lower-limb orthoses, prostheses, and exoskeletons. Med. Eng. Phys. 34, 397–408. 10.1016/j.medengphy.2011.11.018 22177895

[B26] Kirsch N. Alibeji N. Dicianno B. E. Sharma N. (2016). Switching control of functional electrical stimulation and motor assist for muscle fatigue compensation, in 2016 American Control Conference (ACC) (Boston, MA: IEEE), 4865–4870.

[B27] Kobetic R. To C. S. Schnellenberger J. R. Audu M. L. Bulea T. C. Gaudio R.. (2009). Development of hybrid orthosis for standing, walking, and stair climbing after spinal cord injury. J. Rehabil. Res. Dev. 46, 447–462. 10.1682/JRRD.2008.07.0087 19675995

[B28] Lambach R. L. Stafford N. E. Kolesar J. A. Kiratli B. J. Creasey G. H. Gibbons R. S.. (2018). Bone changes in the lower limbs from participation in an fes rowing exercise program implemented within two years after traumatic spinal cord injury. J. Spinal Cord Med. 1–9. 10.1080/10790268.2018.1544879 30475172 PMC7241570

[B29] Liu M. Q. Anderson F. C. Schwartz M. H. Delp S. L. (2008). Muscle contributions to support and progression over a range of walking speeds. J. Biomech. 41, 3243–3252. 10.1016/j.jbiomech.2008.07.031 18822415 PMC4423744

[B30] Lynch C. L. Popovic M. (2008). Functional electrical stimulation. IEEE Cont. Syst. 28, 40–50. 10.1109/MCS.2007.914689

[B31] Makssoud H. Guiraud D. Poignet P. (2004). Mathematical muscle model for functional electrical stimulation control strategies, in Proceedings of the IEEE International Conference on Robotics and Automation, ICRA'04., Vol. 2 (New Orleans, LA: IEEE), 1282–1287.

[B32] Marion M. S. Wexler A. S. Hull M. L. (2013). Predicting non-isometric fatigue induced by electrical stimulation pulse trains as a function of pulse duration. J. Neuroeng. Rehabil. 10:13. 10.1186/1743-0003-10-13 23374142 PMC3626903

[B33] Martín J. M. R. (2004). Electroterapia en Fisioterapia (Madrid). Ed. Médica Panamericana.

[B34] McDonald M. Kevin-Garrison M. Schmit B. (2005). Length-tension properties of ankle muscles in chronic human spinal cord injury. J. Biomech. 38, 2344–2353. 10.1016/j.jbiomech.2004.10.024 16214482

[B35] Menegaldo L. Fleury A. Weber H. (2006). A cheap optimal control approach to estimate muscles forces in musculoskeletal systems. J. Biomech. 39, 1787–1795. 10.1016/j.jbiomech.2005.05.029 16033695

[B36] Michaud F. Lugris U. Ou Y. Cuadrado J. Kecskemethy A. (2015). Influence of muscle recruitment criteria on joint reaction forces during human gait, in Proceedings ECCOMAS Thematic Conference Multibody Dynamics, paper, Vol. 141 (Barcelona), 1024–1031.

[B37] Nagano A. Gerritsen K. (2001). Effects of neuromuscular strength training on vertical jumping performance - a computer simulation study. J. Appl. Biomech. 17, 113–128. 10.1123/jab.17.2.113

[B38] Nigg B. Herzog W. (1999). Biomechanics of the Musculo-Skeletal System, Vol. 192. New York, NY: Wiley.

[B39] Nightingale E. Raymond J. Middleton J. Crosbie J. Davis G. (2007). Benefits of fes gait in a spinal cord injured population. Spinal Cord 45:646. 10.1038/sj.sc.3102101 17646840

[B40] Obinata G. Fukada S. Matsunaga T. Iwami T. Shimada Y. Miyawaki K. (2007). Hybrid control of powered orthosis and functional neuromuscular stimulation for restoring gait, in 2007 29th Annual International Conference of the IEEE Engineering in Medicine and Biology Society (Lyon: IEEE), 4879–4882. 10.1109/IEMBS.2007.4353433 18003099

[B41] Ojeda J. (2012). Aplicación de las técnicas MBS al sistema locomotor humano. (Ph.D. thesis). Universidad de Sevilla, Seville.

[B42] Ou Y. (2011). An analysis of optimization methods for identifying muscle forces in human Gait. (Ph.D. thesis). University of Duisburg, Essen.

[B43] Pipeleers G. Demeulenaere B. Jonkers I. Spaepen P. Van der Perre G. Spaepen A. (2007). Dynamic simulation of human motion: numerically efficient inclusion of muscle physiology by convex optimization. Optimiz. Eng. 9, 213–238. 10.1007/s11081-007-9010-6

[B44] Pizzolato C. Reggiani M. Modenese L. Lloyd D. (2017). Real-time inverse kinematics and inverse dynamics for lower limb applications using opensim. Comput. Methods Biomech. Biomed. Eng. 20, 436–445. 10.1080/10255842.2016.1240789 27723992 PMC5550294

[B45] Qiu S. Taylor J. A. (2016). Hybrid functional electrical stimulation exercise for improved cardiorespiratory fitness in SCI, in The Physiology of Exercise in Spinal Cord Injury (Boston, MA: Springer), 269–286.

[B46] Rajagopal A. Dembia C. L. DeMers M. S. Delp D. D. Hicks J. L. Delp S. L. (2016). Full-body musculoskeletal model for muscle-driven simulation of human gait. IEEE Trans. Biomed. Eng. 63, 2068–2079. 10.1109/TBME.2016.2586891 27392337 PMC5507211

[B47] Rasmussen J. Damsgaard M. Voigt M. (2001). Muscle recruitment by the min/max criterion—a comparative numerical study. J. Biomech. 34, 409–415. 10.1016/S0021-9290(00)00191-3 11182135

[B48] Rengifo C. Aoustin Y. Plestan F. Chevallereu C. (2010). Distribution of forces between synergistics and antogonistics muscles using an optimization criterion depending on muscle contraction behaviour. J. Biomech. Eng. 132, 1–11. 10.1115/1.4001116 20387972

[B49] Riener R. Quintern J. Schmidt G. (1996). Biomechanical model of the human knee evaluated by neuromuscular stimulation. J. Biomech. 29, 1157–1167. 10.1016/0021-9290(96)00012-7 8872272

[B50] Seth A. Hicks J. L. Uchida T. K. Habib A. Dembia C. L. Dunne J. J.. (2018). Opensim: simulating musculoskeletal dynamics and neuromuscular control to study human and animal movement. PLoS Comput. Biol. 14:e1006223. 10.1371/journal.pcbi.1006223 30048444 PMC6061994

[B51] Sharma N. Mushahwar V. Stein R. (2014). Dynamic optimization of fes and orthosis-based walking using simple models. IEEE Trans. Neural Syst. Rehabil. Eng. 22, 114–126. 10.1109/TNSRE.2013.2280520 24122568

[B52] Stewart A. M. Pretty C. G. Adams M. Chen X. (2017). Review of upper limb hybrid exoskeletons. IFAC-PapersOnLine 50, 15169–15178. 10.1016/j.ifacol.2017.08.2266

[B53] Tepavac D. Schwirtlich L. (1997). Detection and prediction of fes-induced fatigue. J. Electromyogr. Kinesiol. 7, 39–50. 10.1016/S1050-6411(96)00008-9 20719690

[B54] Vromans M. Faghri P. D. (2018). Functional electrical stimulation-induced muscular fatigue: effect of fiber composition and stimulation frequency on rate of fatigue development. J. Electromyogr. Kinesiol. 38, 67–72. 10.1016/j.jelekin.2017.11.006 29169055

[B55] Watanabe T. Futami R. Hoshimiya N. Handa Y. (1999). An approach to a muscle model with a stimulus frequency-force relationship for fes applications. IEEE Trans. Rehabil. Eng. 7, 12–18. 10.1109/86.750545 10188603

[B56] Yamaguchi G. T. (2005). Dynamic Modeling of Musculoskeletal Motion: A Vectorized Approach for Biomechanical Analysis in Three Dimensions. New York, NY: Springer Science & Business Media.

[B57] Yan T. Cempini M. Oddo C. M. Vitiello N. (2015). Review of assistive strategies in powered lower-limb orthoses and exoskeletons. Robot. Auton. Syst. 64, 120–136. 10.1016/j.robot.2014.09.032

[B58] Young A. J. Ferris D. P. (2017). State of the art and future directions for lower limb robotic exoskeletons. IEEE Trans. Neural Syst. Rehabil. Eng. 25, 171–182. 10.1109/TNSRE.2016.2521160 26829794

[B59] Zajac F. (1989). Muscle and tendon: properties, models, scaling and applications to biomechanics and motor control. Crit. Rev. Biomed. Eng. 17, 359–411. 2676342

